# A Novel Inactive Isoform with a Restored Reading Frame Is Expressed from the Human Interferon Lambda 4 TT Allele at rs368234815

**DOI:** 10.1089/jir.2022.0199

**Published:** 2023-09-15

**Authors:** Seema Bharatiya, Aditya Agarwal, Sreedhar Chinnaswamy

**Affiliations:** ^1^ Infectious Disease Genetics, National Institute of Biomedical Genomics, Kalyani, West Bengal, India.; ^2^ Regional Centre for Biotechnology, Faridabad, India.

**Keywords:** IFNL4, IFN-λ4, rs368234815, type III IFNs, splicing isoforms

## Abstract

The TT allele of the dinucleotide variant rs368234815 (TT/ΔG) abolishes the open reading frame (ORF) created by the ancestral ΔG allele of the human interferon lambda 4 (*IFNL4*) gene, thus preventing the expression of a functional IFN-λ4 protein. While probing the expression of IFN-λ4 in human peripheral blood mononuclear cells (PBMCs), using a monoclonal antibody that binds to the C-terminus of IFN-λ4, surprisingly, we observed that PBMCs obtained from TT/TT genotype individuals could also express proteins that reacted with the IFN-λ4-specific antibody. We confirmed that these products did not emanate from the *IFNL4* paralog, IF1IC2 gene. Using cell lines and overexpressing human *IFNL4* gene constructs, we obtained evidence from Western blots to show that the TT allele could express a protein that reacted with the IFN-λ4 C-terminal-specific antibody. It had a molecular weight similar if not identical to IFN-λ4 expressed from the ΔG allele. Furthermore, the same start and stop codons used by the ΔG allele were used to express the novel isoform from the TT allele suggesting that a restoration of the ORF had occurred in the body of the mRNA. However, this TT allele isoform did not induce any IFN-stimulated gene expression. Our data do not support a ribosomal frameshift that leads to the expression of this new isoform, implying that an alternate splicing event may be responsible. An N-terminal-specific monoclonal antibody did not react with the novel protein isoform suggesting that the alternate splicing event likely occurs beyond exon 2. The new isoform is glycosylated similar to the functional IFN-λ4 and is also secreted. Furthermore, we show that the ΔG allele can also potentially express a similarly frameshifted isoform. The splicing event that leads to the generation of these novel isoforms and their functional significance remains to be elucidated.

## Introduction

Human interferon lambda 4 (IFNL4 or IFN-λ4) is a newly discovered type III IFN that has been associated with hepatitis C virus and other infections (Prokunina-Olsson et al., 2021, Prokunina-Olsson et al., 2013). Its expression is regulated by a dinucleotide variant rs368234815 (TT/ΔG), in which the ΔG allele allows the formation of an open reading frame (ORF) leading to the expression of a fully functional IFN-λ4 (p179 isoform) and some inactive isoforms such as p107, p131, and p170 (Prokunina-Olsson et al., 2013). The initial characterization of *IFNL4* by Prokunina-Olsson and others showed that the TT allele abolishes the ORF, but can express inactive isoforms such as p124 and p143 that are unrelated to IFN-λ4 (Prokunina-Olsson et al., 2013).

TaqMan primers and probes specifically designed to amplify regions specific to different isoforms of the ΔG allele have been described (Hong et al., 2016), but these can also amplify mRNA generated from the TT allele since translation but not transcription is discriminatory toward the two alleles. The commercial availability of antibodies, raised against p179, has provided additional tools for researchers to probe the expression of this enigmatic protein in different primary and secondary human cells (Chen et al., 2021; Minas et al., 2018; Obajemu et al., 2017).

## Materials and Methods

### Cloning and site-directed mutagenesis

A full-length (exons and introns only) 1.4 kb *IFNL4* gene amplified from a TT/TT (at rs368234815) homozygous individual was cloned in pcDNA3.1+ (Invitrogen) by using primers listed in Supplementary Table S1. A site-directed mutagenesis (SDM) kit (NEB) was used to mutate the TT allele in to ΔG allele in the above construct, without changing any other nucleotides in the rest of the gene. The constructs are referred to as TT or ΔG, hereafter. *IF1IC2*, a 1.5 kb gene [from start to stop codon (including the premature stop codon) with all exons and introns] was similarly cloned from human genomic DNA by using the primers listed in Supplementary Table S1. The construct is referred to as pIF1IC2. Several mutations were introduced into the TT or ΔG either using the SDM kit or by incorporating the mutations/deletions/additions within the primers used for PCR (Supplementary Table S1).

All clones were confirmed by Sanger sequencing of the plasmid DNA. The wild-type (wt) IFN-λ4 cDNA clone, pIFNL4-Halo, was a gift from Ludmila Prokunina-Olsson (NIH), while the codon optimized IFN-λ4 cDNA was commercially synthesized (Genescript, India). The TT mutation-addition was introduced into the wt cDNA clones using SDM. Green fluorescent protein (GFP; Addgene) was cloned in either pCDNA+ vector with or without the ATG start codon. GFP without the ATG start codon was also cloned immediately before the TGA stop codon of TT (in pCDNA+) along with a GGGS linker sequence to express a fusion product with the protein translated from the TT allele.

### Genotyping at rs368234815

Human genomic DNA was extracted from whole blood using a QIAamp Blood DNA Mini Kit (Qiagen). PCR was used to amplify a 1.4 kb amplicon from the genomic DNA, using the primers listed in Supplementary Table S1, which was Sanger sequenced.

### Cell culture and transfection

HEK293, C33A [a gift from Prof. Sharmila Sengupta, National Institute of Biomedical Genomics (NIBMG), Kalyani] (ATCC, USA), and Huh7.5 (a gift from Charles Rice, The Rockefeller University, USA) cells were maintained in Dulbecco's modified Eagle's medium (Himedia) in addition to 10% FBS and penicillin/streptomycin (all from Gibco) at 37°C and 5% CO_2_. Cells were seeded into 6-well [for Western blot (WB)] or 12-well (for qPCR) plates and allowed to reach a 70%–80% confluency (∼24 h). Cells were transfected with pcDNA3.1 or pIF1IC2, TT or ΔG, or the cDNA constructs with Lipofectamine LTX and Plus (Invitrogen). Unless mentioned otherwise, 2 μg/well of the constructs was transfected for WB experiments. Twelve hours (for qPCR) or 24 h (for WB) post-transfection, cells were collected and processed for WB/qPCR.

For fluorescence experiments, HEK293 cells seeded in clear bottom white plates (Corning, costar) were transfected with 100 ng of the different constructs (in pCDNA+) at near confluency and the fluorescence intensity was read after 24 h in the EnSight multimode plate reader (PerkinElmer) using Kaleido data acquisition and analysis program.

### Peripheral blood mononuclear cell isolation

The NIBMG Bioethics Committee approved this study with the certificate No. NIBMG/2022/1/0018. Five milliliters of blood was drawn from volunteers after obtaining informed consent. Peripheral blood mononuclear cells (PBMCs) were isolated using the Ficoll density gradient method (Sigma). Isolated PBMCs were plated or not (fresh PBMCs) in 6-well plates with RPMI Medium-1640 (Gibco) along with 10% FBS and incubated at 37°C and 5% CO_2_. After 5 h, cells were either treated or not for 24 h with phorbol 12-myristate 13-acetate (PMA; Sigma; 100 ng/mL), polyI:C [lower molecular weight (LMW); InvivoGen; at 50 μg/mL], and lipopolysaccharides [LPS (Sigma), at 0.5 μg/mL]. After 24 h, cells were collected and processed for WB.

### RNA isolation, reverse transcription, and qPCR

Total RNA was isolated using the TRIzol reagent (Invitrogen). cDNA was synthesized from 1 μg of RNA using the Verso cDNA Synthesis Kit (Thermo Scientific). qPCR was performed in the QuantStudio 5 (Applied Biosystems) using custom-made isoform-specific IFNL4 TaqMan probes and primers as described previously for the p179, p131, and p107 isoforms, and for the *IFNL3* gene (Hong et al., 2016) using *HPRT*/*ACTB* as the housekeeping control gene; IFN-stimulated genes (ISGs): *MX1*, *OAS1*, *ISG15*, and *IFITM3* were measured using SYBR Green after amplifying the cDNA using the primers described previously (Supplementary Table S1). *GAPDH* was used as an internal control. Relative fold change in expression was calculated using the 2^−ΔΔ^CT method.

### Western blotting

The proteins were run on 15% SDS-PAGE gels, and WB analysis of human recombinant IFN-λ4 was carried out using the rabbit anti-IFN-λ4 (RAB, rabbit monoclonal, 1:2,000, ab196984; Abcam) or mouse anti-IFN-λ4 (MAB, mouse monoclonal, 1:500, MABF227; Millipore) or anti-HA (HA, rabbit monoclonal, 1:1,000, C2954; CST). Following overnight treatment with the primary antibody, the blots were developed with a secondary HRP-linked goat anti-rabbit antibody (Millipore; 1:1,000 dilution) or goat anti-mouse HRP-conjugated secondary antibody (Millipore; 1:1,000 dilution). For beta-actin (Santa Cruz Biotechnology), primary antibodies at 1:10,000 dilution were used. The blots were developed and imaged in ChemiDoc MP imaging system (Bio-Rad).

### Deglycosylation

Deglycosylation was done by using PNGase F (NEB; P0705S). For deglycosylation, 9 μL of cell lysate along with 1 μL of 10 × denaturing buffer was heated at 100°C for 10 min. After denaturation, 2 μL of glyco buffer, 2 μL of 10% NP-40, 1 μL of PNGase F, and 5 μL of deionized water were added. This mixture was incubated at 37°C overnight and then proceeded with WB.

### Trichloroacetic acid precipitation

Trichloroacetic acid was added to the clarified media in 1:4 ratio and incubated at 4°C for 30 min. The sample was centrifuged at 14,000 rpm for 10 min at 4°C. The supernatant was decanted, and the precipitated protein pellet was washed thrice with 200 μL of cold acetone by centrifugation at 14,000 rpm for 5 min at 4°C. Pellet was dried at 95°C for 5 min. Lysis buffer was added to the pellet and proceeded to WB.

## Results

### PBMCs from individuals with TT/TT genotype at rs368234815 express proteins that react with IFN-λ4-specific antibodies

**FIG. 1.**
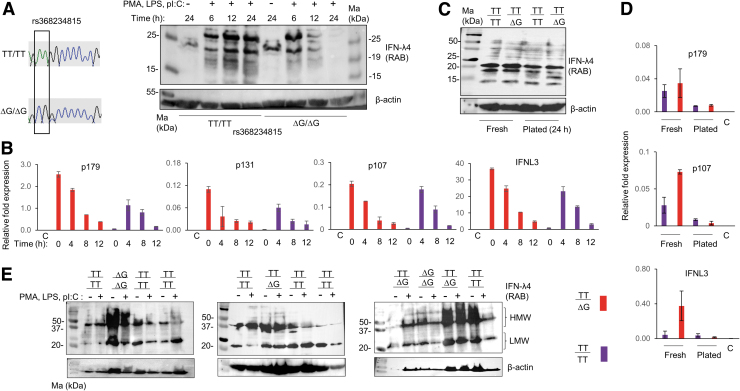
IFN-λ4-like proteins are expressed in both TT/TT and ΔG/ΔG genotype individuals. **(A)** (*left*) Chromatogram showing the Sanger sequencing genotyping for rs368234815 in two individuals. (*right*) WB performed on PBMC lysates with the RAB (Cat. No. ab196984; Abcam) either with or without treatment as detailed in the “Materials and Methods” section, for different time points as shown. **(B)** Results of qPCR carried out from fresh (0 h) or stimulated PBMCs for the indicated time points from a TT/TT and TT/ΔG genotype individual. RNA isolated from PBMCs was subject to RT-qPCR using specific TaqMan reagents described in Hong et al. (2016) using *HPRT* as the housekeeping control. RNA from untreated HEK293 cells grown for 24 h was used as negative control. Stimulation was done to plated PBMCs for the given time points as in **(A)**. **(C)** WB from fresh and plated PBMCs from TT/TT and TT/ΔG genotype individuals (different from those shown in **B**) using RAB; the plated cells (without any stimulation) were incubated for 24 h before RNA isolation or lysate preparation. **(D)** RT-qPCR for the samples shown in **(C)**. **(E)** WB from lysates of unstimulated (plated for 24 h) or stimulated (for 24 h, as in **A**) PBMCs of different individuals. Seven out of the 12 individuals were TT/TT, but still expressed proteins that reacted with the RAB raised specifically against the C-terminus of IFN-λ4. HMW-high molecular weight, LMW-low molecular weight; C- HEK293 cell RNA control. For qPCR experiments, mean values from two technical replicates are shown, error bars depict SD. IFN-λ4, interferon lambda 4; PBMCs, peripheral blood mononuclear cells; RT, reverse transcription; WB, Western blot.

In the beginning of this study, we were interested to look for the expression of IFN-λ4 in human PBMCs. For the initial experiment, we identified two individuals who were homozygous for the major (TT) and minor (ΔG) alleles (Fig. 1A) at rs368234815 and isolated their PBMCs. The PBMCs were cultured for different time points either with or without treatment with a TLR-3 agonist polyI:C, a TLR-4 agonist LPS, and an NF-kB activator PMA.

After incubation, the cells were lysed and probed in WB with a rabbit monoclonal antibody (RAB) that is raised against the C-terminus of IFN-λ4, and that reacts only against the human IFN-λ4 protein by recognizing amino acids 128–152 (Paquin et al., 2016). [We had to standardize these experiments since we saw that the routine concentration of cell lysates (1–50 μg protein/well, BioRad protocols) used in WBs failed to show any reactive bands. However, using 80–100 μg of protein from PBMC lysates proved beneficial, suggesting that a tiny fraction of the PBMCs may be producing these proteins, data not shown.] Surprisingly, we saw that TT/TT samples were also expressing proteins that reacted with the IFN-λ4-specific antibody at similar positions to those expressed by ΔG/ΔG samples, only their kinetics of expression being different (Fig. 1A).

We were interested to assess mRNA levels specific to *IFNL4* in the PBMCs. We used PBMCs from a TT/ΔG and a TT/TT individual to examine this. We tested the mRNA expression of all the three isoforms of *IFNL4* (p179, p131, and p107) and of *IFNL3* in both freshly isolated PBMCs (without plating) and PBMCs that were cultured and treated, as in Fig. 1A, for several time points. The TaqMan probes and primers described earlier (Hong et al., 2016) that specifically amplify different *IFNL4* isoforms were used (Supplementary Fig. S1A). We used RNA isolated from cultured HEK293 cells as negative control. The TT/ΔG sample showed high induction of all the isoforms of *IFNL4* and of *IFNL3* even in the untreated PBMCs (Fig. 1B). Treatment with PMA, LPS, and polyI:C led to no further induction of any of the gene products in this individual.

However, treatment with the above stimulants led to a strong induction of all the gene products in the TT/TT sample (Fig. 1B). There was a sharp decline of the stimulatory effect by 12 h of treatment in both the samples. Nonetheless, these results show that *IFNL4* mRNA is expressed in human PBMCs, regardless of the dinucleotide variant rs368234815. Since, we saw *IFNL4* mRNA expressed even without any stimulation in the heterozygote individual, we tested if it is true at the protein level. When we probed lysates made from fresh PBMCs with the RAB, we saw again that RAB reactive proteins were expressed from the TT/TT individual that were indistinguishable from the TT/ΔG sample (Fig. 1C). Plating the PBMCs for 24 h (without any treatment) decreased the intensity of these products. The mRNA expression pattern mostly agreed with the WB results (Fig. 1D).

Next, we identified more individuals with the TT/TT genotype at rs368234815 (Supplementary Fig. S1B) and cultured their PBMCs for 24 h with or without treatment as before and probed the lysates in WB using the RAB. Even though the effect of the stimulation was variable across samples in this experiment, it was clear that there were RAB reactive proteins that could be detected even in TT/TT samples similar to the TT/ΔG or ΔG/ΔG samples (Fig. 1E). There were two forms of the RAB reactive proteins: one that moved around 37–50 kDa [higher molecular weight (HMW)] and those that moved around 20–25 kDa (LMW). The HMW bands seem to originate from CD14+ monocytes (Supplementary Fig. S1C).

### Characterization of a new isoform expressed from the TT allele of IFNL4 that reacts with the IFN-λ4-specific antibody

The results in Fig. 1 suggest to us the following. (1) It is possible that the proteins that we see in TT/TT samples could be due to the nonspecific reactivity of the RAB; (2) if (1) above is true, then a functional IFN-λ4 is not likely expressed in the samples of the ΔG allele carriers, or (3) A paralog of human *IFNL4* may be the origin of these bands. To address point (1) above, we subjected the fresh PBMC lysates from all three genotype individuals to WBs using a mouse monoclonal antibody (MAB) that binds to the N-terminus of IFN-λ4 (Paquin et al., 2016) (Supplementary Fig. S1D). We saw that the MAB reacted at least with the HMW products if not with the LMW ones.

Therefore, it is unlikely that two different antibodies raised against different regions of the same protein could be producing nonspecific bands in our WBs, at least not with regard to the HMW products coming from the TT/TT samples. Next, to address point (3) above, when we queried, we saw that *IF1IC2* or *IFNL4P1*, a pseudogene that is present upstream of *IFNL2*, which may be a duplicated gene that emanated from *IFNL4* (Wack et al., 2015) (Supplementary Fig. S2), showed a strong match with the antigen used to raise the RAB (Prokunina-Olsson et al., 2013). Even though human *IFNL4P1* is described as a pseudogene due to the premature termination of translation by a TAA stop codon early in the ORF, a recent study (Premzl, 2020) has speculated, based on the splicing pattern seen in other species, that the premature stop codon could be missed if splicing occurs at one of the first “GT-AG” splice-site junctions (Abramowicz and Gos, 2018), leading to the expression of a 169 residue-containing IF1IC2 protein (Supplementary Fig. S2) that could potentially react with the RAB.

**FIG. 2.**
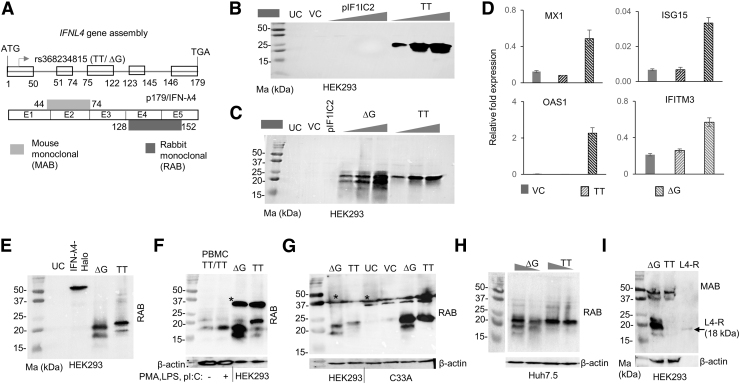
TT allele expresses a novel p179-like isoform. **(A)** The *top* schematic shows the *IFNL4* with exons and introns (numbers at *bottom* of exons indicate the amino acid positions in the protein); the entire gene was cloned in a pcDNA3.1+ vector from start to the stop codons retaining all exons and introns, the constructs are referred to as TT or ΔG depending on the allele they carry at rs368234815. The *bottom* schematic shows the p179 protein with the two antibodies used in the study shown with their binding regions. The number beside the binding regions show the antigen length in terms of amino acid positions used to raise the antibody. E-exon. **(B, C)** The pIF1IC2 (*IF1IC2* full-length gene cloned along with all exons and introns) or TT or ΔG constructs were transfected in HEK293 cells at 0.5, 1, and 2 μg/well (pIF1IC2 at 1 μg/well in **C**) for 24 h before performing WB with RAB. **(D)** RT-qPCR of ISGs expressed from VC (pCDNA) or TT or ΔG transfected HEK293 cells incubated for 24 h. *GAPDH* was used as a housekeeping gene. The data are representative of at least two independent experiments; mean value from three technical replicates is shown with error bars depicting SD. **(E)** WB shows that TT allele expresses a novel RAB reacting protein. p179 expressing cDNA clone pIFNL4-Halo (∼54 kDa fusion protein) was used to check the specificity of the RAB. **(F)** PBMCs from a unstimulated or stimulated (for 24 h, as in Fig. 1A) TT/TT individual were run in parallel to lysates from TT or ΔG allele overexpressing HEK293 cells. The TT and ΔG constructs were overexpressed in C33A cells **(G)** or Huh7.5 cells **(H)** (constructs were expressed at 1 and 2 μg/well in **H**) and lysates were probed with RAB. The symbol * in **(F)** and **(G)** indicates HMW products expressed that are unrelated to overexpressed TT or ΔG constructs. **(I)** The novel isoform from TT allele may not have an N-terminus such as p179. The MAB (MABF227; Millipore) that binds to exons 1 and 2 as shown in **(A)** could not bind to the novel isoform expressed from the TT construct in WB (also shown in Supplementary Fig. S3C). L4-R, a recombinant truncated IFN-λ4 protein from R&D Biosystems that runs at 18 kDa (shown by the *arrow*) (catalog No. 9165-IF). UC-untransfected cells; VC-vector (pcDNA3.1) control transfected at 1 μg/well; the names of the cell lines used in different blots are shown below each blot. ISGs, IFN-stimulated genes.

To test this possibility, we cloned the full-length *IF1IC2* gene in a pcDNA3.1+ (Invitrogen) expression vector; similarly, we constructed another clone, pIFNL4 (TT) (referred to as TT hereafter), that contained the full-length *IFNL4* gene (from start to stop codon, including all introns) obtained from a TT/TT homozygous individual. We used two different well-characterized (Paquin et al., 2016) monoclonal antibodies to probe the gene products obtained from these constructs overexpressed in different cell lines (Fig. 2A). When we transfected the constructs in to HEK293 cells and performed WB with the RAB, the results we obtained were surprising. We saw no reactive bands from the pIF1IC2 construct overexpressed cells, but we detected an ∼20–25 kDa protein from the TT construct, whose intensity depended on the amount of DNA that was transfected (Fig. 2B).

Next, we mutated the TT allele from the pIFNL4 (TT) construct to the ΔG allele (referred to as ΔG hereafter) and repeated the experiment along with the TT allele construct (Fig. 2C). Only the ΔG allele but not the TT allele gave rise to distinct lower sized bands, however, both alleles expressed the 20–25 kDa size proteins seen in Fig. 2B. The products expressed from the TT allele do not activate any ISGs (Fig. 2D). In some experiments in HEK293 cells, we saw that the mobility of the RAB reactive bands from the TT and ΔG constructs differed slightly (Fig. 2E).

We simultaneously ran products from a PBMC sample obtained from a TT/TT individual along with the products from overexpressed TT or ΔG constructs in HEK293 cells and saw that at least some of the bands expressed from the ΔG construct moved at similar positions to that from the TT/TT PBMC sample (Fig. 2F). We also confirmed that the TT construct can express RAB reactive proteins in other cell lines, first in a cervical cancer cell line C33A and then in a liver-origin cell line Huh7.5 (Fig. 2G, H). In some of the experiments, we saw that all the three cell lines HEK293, Huh7.5 (in vector-transfected cells, but not in untransfected cells), and C33A (both in untransfected and vector-transfected cells) (Supplementary Fig. S3A, B) could express HMW products (marked as * in Fig. 2F, G) that reacted with the RAB unrelated to the pIFNL4 construct overexpression, but their appearance was inconsistent across experiments.

Interestingly, these HMW products reacted with the MAB, similar to the results that we saw from PBMC experiments (Supplementary Figs. S1D and S3B). Since all three cell lines were heterozygous for the rs368234815 genotype (not shown), it is difficult to say that some of these HMW bands are indeed expressed from the TT allele. Interestingly, the bands specific to the overexpressed TT construct in HEK293 cells did not react with the MAB that binds to peptides translated from exon 1 and 2 (Fig. 2A, I and Supplementary Fig. S3C). This would suggest that the N-terminus of the protein is not exactly like wt p179/IFN-λ4.

### The novel isoform from the TT allele likely arises from an alternate splicing event

**FIG. 3.**
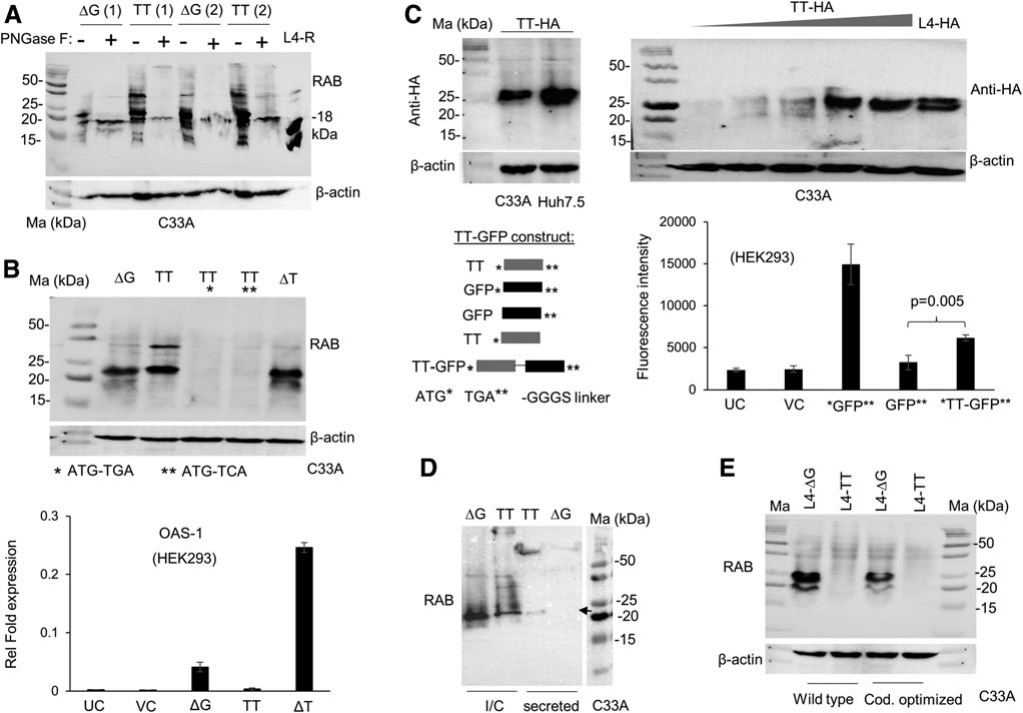
The novel isoform from TT allele is glycosylated and secreted. **(A)** TT and ΔG constructs at two concentrations (1 and 2 μg/well as indicated *above* the lanes) were transfected into C33A cells and incubated for 24 h, after which lysates were prepared and were treated or not with PNGaseF, as detailed in the “Materials and Methods” section. WB was performed with RAB as before. L4-R, 25 ng of a recombinant IFN-λ4 protein from R&D Biosystems that runs at 18 kDa (catalog No. 9165-IF), was run as a control. **(B)** The novel isoform is expressed from the same start codon as p179. (*top*) WB showing the products from overexpressed wt ΔG or wt/mutant TT constructs. The start codon was mutated to other codons as shown *below* the blots; ΔT, ie, deletion of a single T from TT results in restoration of ORF and hence a fully functional p179 with an Ala-Val mutation at 22nd amino acid is translated. (*bottom*) qPCR results for OAS1 gene from HEK293 cells transfected with the different constructs shown. Average of means from two independent experiments carried out in technical triplicates is shown with error bars showing SD of the two averages. **(C)** (*top*) A HA tag was introduced before the stop codon in the TT construct and transfected in C33A or Huh 7.5 cells (*left*); an increasing concentration (0.5, 1.0, 1.5, 2.0, and 2.5 μg/well) of the construct along with a wt IFN-λ4 expressing cDNA with a C-terminal HA tag (L4-HA at 1 μg/well) was transfected in to C33A cells and probed with an anti-HA antibody in WB (*right*); (*bottom*) fluorescence intensity of a fusion GFP with the product expressed from the TT allele in HEK293 cells. UC-untransfected cells; VC-vector control; GFP**: GFP without a start codon cloned in pCDNA3.1; *TT-GFP**: GFP cloned in frame with the TT allele just before the stop codon along with a GGGS linker sequence (*left* schematic). The mean reading from biological triplicates is shown with error bars depicting SD. *P*-value was calculated using two-tailed Student *t*-test for independent means. **(D)** The novel isoform of TT allele is secreted more efficiently than the ΔG allele products in C33A cells. Equal amounts of the TT or ΔG constructs were transfected, and lysates or TCA precipitated supernatants probed in WB are shown. *Arrow* shows the secreted product from the TT construct carrying cell supernatants. I/C-intracellular. **(E)** Ribosomal frame shifting is not the mechanism behind generation of the novel isoform from the TT allele. cDNA clones expressing IFN-λ4 with a wt sequence or codon optimized sequence were introduced with the TT mutation at rs368234815. The ΔG or TT allele carrying cDNA constructs (cloned in pcDNA3.1 vectors) transfected at 1 μg/well were transfected and cell lysates were probed by WB. The antibodies used in WB are shown next to the blots and the cell lines used for transfection are also shown below the blots. GFP, green fluorescent protein; ORF, open reading frame; TCA, trichloroacetic acid.

IFN-λ4 is known to be heavily glycosylated (Chen et al., 2021). We were interested to see if the novel isoform from the TT allele is glycosylated and/or secreted. We overexpressed the ΔG and TT constructs in C33A cells at two different concentrations and subjected the lysates to PNGaseF treatment (Fig. 3A). Similar, to the products from the ΔG construct, the products from the TT construct were also affected in mobility after the treatment. The PNGaseF-treated products from the TT construct migrated very closely to the products from the ΔG construct and with a recombinant IFN-λ4 protein of 18 kDa (R&D Biosystems) (Fig. 3A). We tested if the novel isoform from the TT construct used the same start and stop codons similar to the p179 product from the ΔG allele. For this, we introduced mutations into the start codon or added an HA tag before the stop codon.

The results show that the novel TT allele isoform cannot express an RAB reacting protein if the start codon is mutated, and it also produces an HA tag suggesting the translation of an in-frame protein beyond exons 4 and 5 (Fig. 3B top, 3C top). Furthermore, the TT allele could express a GFP (even though at ∼41% levels to that of GFP-positive control) that lacked a start codon of its own but that which was cloned immediately before the stop codon of *IFNL4* so that the GFP could be expressed only as a fusion protein along with the product of the TT allele (Fig. 3C bottom). When we deleted one T from the TT allele, thus restoring the disrupted frame at rs368234815, we saw the expression of an ISG stimulating RAB reacting IFN similar to the wt p179 expressed from the ΔG allele (Fig. 3B).

Furthermore, the novel isoform is secreted more efficiently than the products of the ΔG allele in C33A cells, but is secreted to a similar extent in Huh7.5 cells (Fig. 3D and Supplementary Fig. S4A).

The above results clearly indicate that a novel protein is produced from the *IFNL4* TT allele that has a restored reading frame beyond the dinucleotide DNA polymorphism rs368234815. Restoration of the reading frame could be due to a ribosomal frameshift, or it could be due to a hitherto unreported splicing event in the body of the mRNA that leads to the reading frame restoration. To address both possibilities, we introduced the TT mutation-addition to the ΔG allele in a p179 expressing cDNA construct. We used two different cDNA constructs, one the wt and another a codon optimized version. However, no RAB reactive products were produced in either of the TT allele carrying versions of the overexpressing cDNA constructs (Fig. 3E). This would suggest no role for a ribosomal frameshift happening during translation of the TT allele mRNA to produce the novel isoform.

**FIG. 4.**
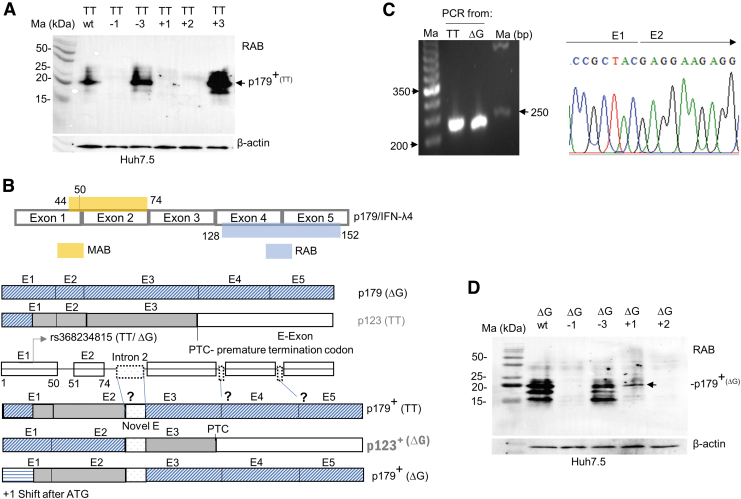
An alternate splice event likely involving introns 2, 3, or 4 may be responsible for generation of the novel isoform from the TT allele. **(A)** WB with RAB of C33A cells transfected with the wt or frameshift mutants of the TT allele is shown. The novel frames generated from the mutations are shown in Supplementary Figure S4B. **(B)** A possible explanation for the alternate splice event that generates a novel TT allele isoform is schematically depicted; the binding sites for the two antibodies are shown as amino acid positions; exon 1 ends at 50th amino acid position. The novel isoform could be generated due to the splicing in of a novel exon (novel E) from the introns leading to a frame restoring change and expression of p179+ from the TT allele. A similar event happening with the ΔG allele will lead to p123^+^, but this may lead to nonsense-mediated decay due to the generation of a premature termination codon as a result of the frameshift. The potential expression of p123^+^ from the ΔG allele can be tested by constructing *a* + 1 frameshift that generates a p179+ in the ΔG allele background as shown. **(C)** The frame shifting alternate splice event may not involve intron 1. PCR products run on ethidium bromide-stained agarose gels amplified from cDNA involving overexpressed TT or ΔG alleles in HEK293 cells using primers that bind to the beginning of exon 1 and the end of exon 2 (Supplementary Table S1; a product size of 223 bp is expected) (*left*); a chromatogram showing the sequence between exon 1–2 junction of PCR products generated from TT allele (*right*); a lack of any overlapping peaks in the junction suggests the absence of any alternate spliced transcripts involving this region. **(D)** Expression of p179+ from the ΔG allele. WB showing the products generated from overexpressed wt or mutant ΔG allele constructs. *A* + 1 frameshift should generally disrupt the frame and abolish the production of a protein that reacts with RAB, but in a frame shifting event, a product that reacts with the RAB, p179+, is produced. Huh7.5 cells were used for experiments in **(A)** and **(D)**.

To understand the origin of the novel TT allele isoform, we introduced frame changing mutations next to the start codon and subjected the overexpressed products to WBs using the RAB (Fig. 4A). These introductions generate premature stop codons at different positions in the reading frame as shown in Supplementary Fig. S4B. Our results agreed with all the predicted changes in the termination of the frame with RAB reactive inactive products seen only with −3 and +3 changes (Fig. 4A and Supplementary Fig. S4C). The alternate splicing event that leads to the frameshift could happen in the junction between any of the exons (Fig. 4B).

However, our results from Fig. 2I (and Supplementary Fig. S3C) suggest that the frameshift event happens beyond exon 2 as the MAB fails to bind to the novel isoform (Figs. 2I and 4B). Indeed, sequencing of a PCR product from cDNA generated from TT allele overexpressed HEK293 cells failed to show any evidence of an alternate splice event in between the exon 1–2 junction (Fig. 4C). Therefore, the frame shifting alternate splice event likely occurs later in the body of the mRNA, which remains to be explored. The generation of the novel “p179^+^” protein from the TT allele also raises the possibility of a similar event happening with the ΔG allele (isoform referred to as p123^+^; Fig. 4B).

This hypothesis could be tested to see if an RAB reacting product is generated from *a* + 1 frame-shifted ΔG allele construct (Fig. 4D); indeed, we saw an RAB reactive protein expressed by the +1 frame-shifted ΔG allele construct (Fig. 4D). These results indicate that a frameshift event could happen during transcription and translation of the human *IFNL4* gene regardless of the alleles present at rs368234815, leading to the generation of novel transcripts. PCR and qPCR products generated with cDNA from TT and ΔG allele overexpressed cells were indistinguishable between the two alleles (Supplementary Fig. S5A, B); sequencing of the PCR products from the TT allele did not identify any new isoforms (Supplementary Fig. S5A, table); a deeper sequencing from shotgun cloned PCR products and/or intron insertion experiments may be needed to identify the contours of the novel isoform.

## Discussion

At least 10 mRNA isoforms, 5 each from the ΔG and TT alleles of human *IFNL4* gene, were identified by RNA-seq and the subsequent molecular analysis (Prokunina-Olsson et al., 2013). There is strong genetic evidence for the causal role for the ΔG allele in hepatitic C virus (Onabajo et al., 2019) and other infections (Prokunina-Olsson et al., 2021). However, establishing the expression of IFN-λ4 in human biopsy samples has been challenging thus far. The results of our study show for the first time that a novel isoform of the TT allele that is similar in sequence to p179, likely downstream from exon 2, could be further confounding such studies.

We began this study with an aim to examine the expression of IFN-λ4 in human PBMCs (Fig. 1). Our observations that TT allele carriers can express RAB reactive proteins led to the design of several molecular biology experiments using overexpressing ΔG and TT allele constructs (Figs. 2–4), in which we characterize a novel isoform of the TT allele likely with a similar sequence to p179 after the proximal end of the protein. The fact that we saw evidence for the expression of such a novel isoform in the PBMCs of TT/TT individuals even under unstimulated conditions (Fig. 1 and Supplementary Fig. S1C) argues against a spurious and experimentally induced production of the novel isoform due to overexpression conditions in cell lines.

A recent report has shown that *IFNL4* mRNA is the most expressed IFN mRNA in bone marrow stem cells of healthy individuals (Gadalla et al., 2020); thus, it is possible that the proteins in our PBMC WBs are from rare stem cell populations since we had to load an unusually large amount of lysate (> 50 μg) to detect them. Furthermore, it remains to be seen whether the novel isoform that we characterize in this study from the TT allele could also have some functional significance, since it is also glycosylated and sometimes more efficiently secreted than IFN-λ4 (Fig. 3D and Supplementary Fig. S4A).

## Supplementary Material

Supplemental data

Supplemental data

Supplemental data

Supplemental data

Supplemental data

Supplemental data
