# Supplementary material for: A Novel Inactive Isoform with a Restored Reading Frame Is Expressed from the Human Interferon Lambda 4 TT Allele at rs368234815
Source: J Interferon Cytokine Res. 2023 Sep 15;43(9):370–8. doi: 10.1089/jir.2022.0199 (PMC10517323; doi:10.1089/jir.2022.0199)
Supplement: Supplemental data [file Suppl_TableS1.docx]

| *MX1* | 5’-GGACATCACTGCTCTCATGC-3’ | 5’-TTTATGGCCTTCTTGAAAATTG-3’ |
| --- | --- | --- |
| *ISG15* | 5’-TCCTGCTGGTGGTGGACAA-3’ | 5’-TTGTTATTCCTCACCAGGATGCT-3’ |
| *OAS1* | 5’-AGAAATACCCCAGCCAAATCTCT-3’ | 5’-TGAGGAGCCACCCTTTACCA-3’ |
| *IFITM* | 5’-CTGGGCTTCATAGCATTCGCCT-3’ | 5’-AGATGTTCAGGCACTTGGCGGT-3’ |
| *GAPDH* | 5’-GGGTGTGAACCATGAGAAGTA-3’ | 5’-GGTGCAGGAGGCATTGCT-3’ |

**Suppl. Table 1:** **List of primers used in this study.**

- **For ISGs tested in the study**

- **TaqMan primers and probes used in this study**

| **Gene** | **Forward primer sequence** | **Reverse primer sequence** | **Probes** |
| --- | --- | --- | --- |
| *IFNL4P179* | CGATCCTGGAGCTGCTG | TTTGTGACGCCTCTTCTGG | CAAGGCAGGCCGCCACATC |
| *IFNL4P131* | CAACTGCTCCTTCCGCC | GGGTTTGTGACGCCTCTTC | CGGCCATCGCTTGAGCTGG |
| *IFNL4P107* | CTCTCGCACTACCGCTC | TTTGTGACGCCTCTTCTGG | CGCTGAGGGACCGCTACCTTG |
|  |  |  |  |

| *IFNL3* | Invitrogen, assay Id Hs04193049_gH |
| --- | --- |

|  |  |  |  |
| --- | --- | --- | --- |

| *ACTB* | Invitrogen, assay Id Id Hs01060665_g1 |
| --- | --- |
| *HPRT1* | Invitrogen, assay Id Hs02800695_m1 |

- **Primers used to generate Site directed mutations (SDM)**

| **SDM Clones** | **Forward primer sequence** | **Reverse primer sequence** |
| --- | --- | --- |
| *Cod. Opt. IFNL4 SDM DG>TT* | 5’GTCATTGCGGAAGGCACCACGG-3’ | 5’-GGTGCATAATACCCAAAGG-3’ |
| *SDM_IFNL4_TT>T* | 5’-AGGCCCCCCGGCGCTGCC-3’ | 5’CTGCGATCACCGTGCACAGGACCCACAGC-3’ |
| *SDM_p179_DG >TT* | 5'-GTGATCGCAGAAGGCCCCCCGG-3' | 5'-CGTGCACAGGACCCACAG-3' |

- **Primers used to generate Mutations in or near start/stop codons.**

| **Mutant Clones** | **Forward primer sequence** | **Reverse primer sequence** |
| --- | --- | --- |
| *IFNL4_1.4Kb_plus1* | 5'-GATATCAAGCTTATGTCGGCCGAGTGTCTGGGCC-3' | 5’-GATATCTCTAGATCAGAGGCAAGGCCCAGAG-3’ |
| *IFNL4_1.4Kb_plus2* | 5'-GATATCAAGCTTATGTCCGGCCGAGTGTCTGGGCC-3' | 5’-GATATCTCTAGATCAGAGGCAAGGCCCAGAG-3’ |
| *IFNL4_1.4Kb_plus3* | 5'-GATATCAAGCTTATGTCACGGCCGAGTGTCTGGGCC-3' | 5’-GATATCTCTAGATCAGAGGCAAGGCCCAGAG-3’ |
| *IFNL4_1.4Kb_minus1* | 5'-GATATCAAGCTTATGGGCCGAGTGTCTGGGCC-3' | 5’-GATATCTCTAGATCAGAGGCAAGGCCCAGAG-3’ |
| *IFNL4_1.4Kb_minus3* | 5'-GATATCAAGCTTATGCCGAGTGTCTGGGCC-3' | 5’-GATATCTCTAGATCAGAGGCAAGGCCCAGAG-3’ |
| *IFNL4_1.4Kb_stop* | 5’GATATCAAGCTTTGACGGCCGAGTGTCTGG-3` | 5’-GATATCTCTAGATCAGAGGCAAGGCCCAGAG-3’ |
| *IFNL4_1.4Kb_ser* | 5’GATATCAAGCTTTCACGGCCGAGTGTCTGG-3’ | 5’-GATATCTCTAGATCAGAGGCAAGGCCCAGAG-3’ |

- **Primers used to generate different clones in this study.**

| **Clones** | **Forward primer sequence** | **Reverse primer sequence** |
| --- | --- | --- |
| *IFNL4_1.4Kb_HA* | 5’GATATCAAGCTTATGCGGCCGAGTGTCTGG-3’ | 5`GATATCTCTAGATCAAGCGTAATCTGGAACATCGTATGGGTAGAGGCAAGGCCCAGAG-3` |
| *Cod. Opt. IFNL4* | 5`GATATCAAGCTTATGCGGCCGTCGGTCTGGGC-3` | 5`-GATATCGGATCCTTAGAGGCAAGGGCCAGAATGTG-3` |
| *Halo_P179* | 5’GATATCAAGCTTATGCGGCCGAGTGTCTGG3’ | 5’GATATCGAATTCTCAGAGGCAAGGCCCAGAG3’ |
| *IFNL4_1.4Kb* | 5’GATATCAAGCTTATGCGGCCGAGTGTCTGG-3’ | 5’-GATATCTCTAGATCAGAGGCAAGGCCCAGAG-3’ |
| *IF1IC2* | 5’GATATCGGTACCATGCGACCGAGTGTGTGGG 3’ | 5’GATATCCTCGAGTCAGAGGCAAGGCCCTGAG 3’ |
| *EGFP W/O start* | 5`GATATCGAATTCGTGAGCAAGGGCGAGGAGCTG-3` | 5`GATATCTCTAGATTATTTGTAGAGCTCGTCCATGC-3` |
| *EGFP* | 5`GATATCGAATTCATGGTGAGCAAGGGCGAGGAGCTG-3` | 5`GATATCTCTAGATTATTTGTAGAGCTCGTCCATGC-3` |
| *TT W/O stop* | 5’GATATCAAGCTTATGCGGCCGAGTGTCTGG-3’ | 5`GATATCGAATTCAGAACCACCTCCGCCGAGGCAAGGCCCAGAGTGTGC-3 |

- **Primers used to amplify and sequence PCR products of cDNA converted *IFNL4* TT/dG transcripts.**

| *Exon1-Exon2* | 5’-ATGCGGCCGAGTGTCTGG-3’ | 5’-CGATGGCCGCGGAGGATC-3’ |
| --- | --- | --- |
| *Exon1-Exon5* | 5’GATATCGAATTCATGCGGCCGAGTGTCTGG3’ | 5’GATATCAAGCTTTCAGAGGCAAGGCCCAGAG3’ |
