# Supplementary material for: A Novel Inactive Isoform with a Restored Reading Frame Is Expressed from the Human Interferon Lambda 4 TT Allele at rs368234815
Source: J Interferon Cytokine Res. 2023 Sep 15;43(9):370–8. doi: 10.1089/jir.2022.0199 (PMC10517323; doi:10.1089/jir.2022.0199)

**S. Fig. 1**:   **A**. The binding regions of the TaqMan primers and probes designed by Hong et al, 2016 to specifically amplify p179, p131 and p107 are shown schematically. Arrows indicate primers and horizontal line indicates the probe. **B.** The chromatograms show the different genotypes at rs368234815 for 12 individuals ( whose PBMCs probed by WBs are shown in Fig. 1E), after Sanger sequencing of PCR amplicons. **C.** WB from freshly isolated PBMCs (not cultured); or separated monocytes (CD14^+^) (after separation from PBMCs of two donors) and the remaining fraction of PBMCs cultured for 24 h separately without any treatment, probed with RAB. The genotypes at rs368234815 of the two volunteers whose PBMCs were used are shown below the blots. Monocytes were isolated with the EasySep TM Human CD14-cell isolation kit (StemCell Technologies). **D.** WB from freshly isolated PBMCs from TT/ ΔG, TT/TT and ΔG/ ΔG genotype (at rs368234815) volunteers probed with RAB and MAB in separate blots. HMW-high mol. wt.; LMW-low mol. wt.

 
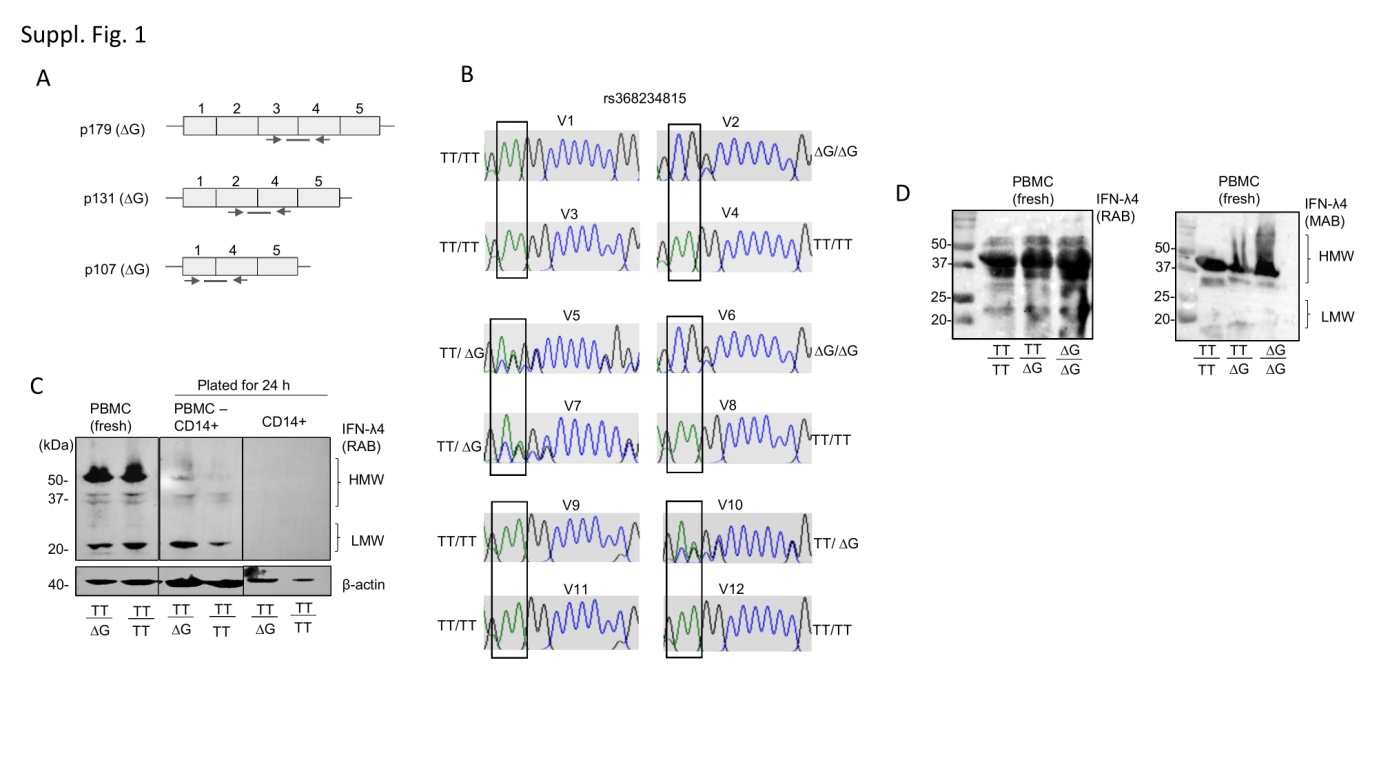

Supplement: Supplemental data [file Suppl_FigureS1.docx]
