# Supplementary material for: A Novel Inactive Isoform with a Restored Reading Frame Is Expressed from the Human Interferon Lambda 4 TT Allele at rs368234815
Source: J Interferon Cytokine Res. 2023 Sep 15;43(9):370–8. doi: 10.1089/jir.2022.0199 (PMC10517323; doi:10.1089/jir.2022.0199)

**S. Fig. 2**: The newly described human *IF1IC2* could be the source of RAB reactive proteins? **A.** Schematic depiction of the *IFNL* locus. **B.** Results from querying the RAB epitope sequence in NCBI Blast (protein to protein). **C.** The alignment of proteins IFNL4 (p179) vs. IF1IC2 and the RAB binding peptide sequence. **D and E.** The *IF1IC2* (**D**) and *IFNL4* (**E**) gene assemblies are shown. The exons are shaded grey while the start, stop and the splice sites (GT-AG) are in bold and italicized; the premature stop codon TAA in *IF1IC2* is also shown in **D**.

 
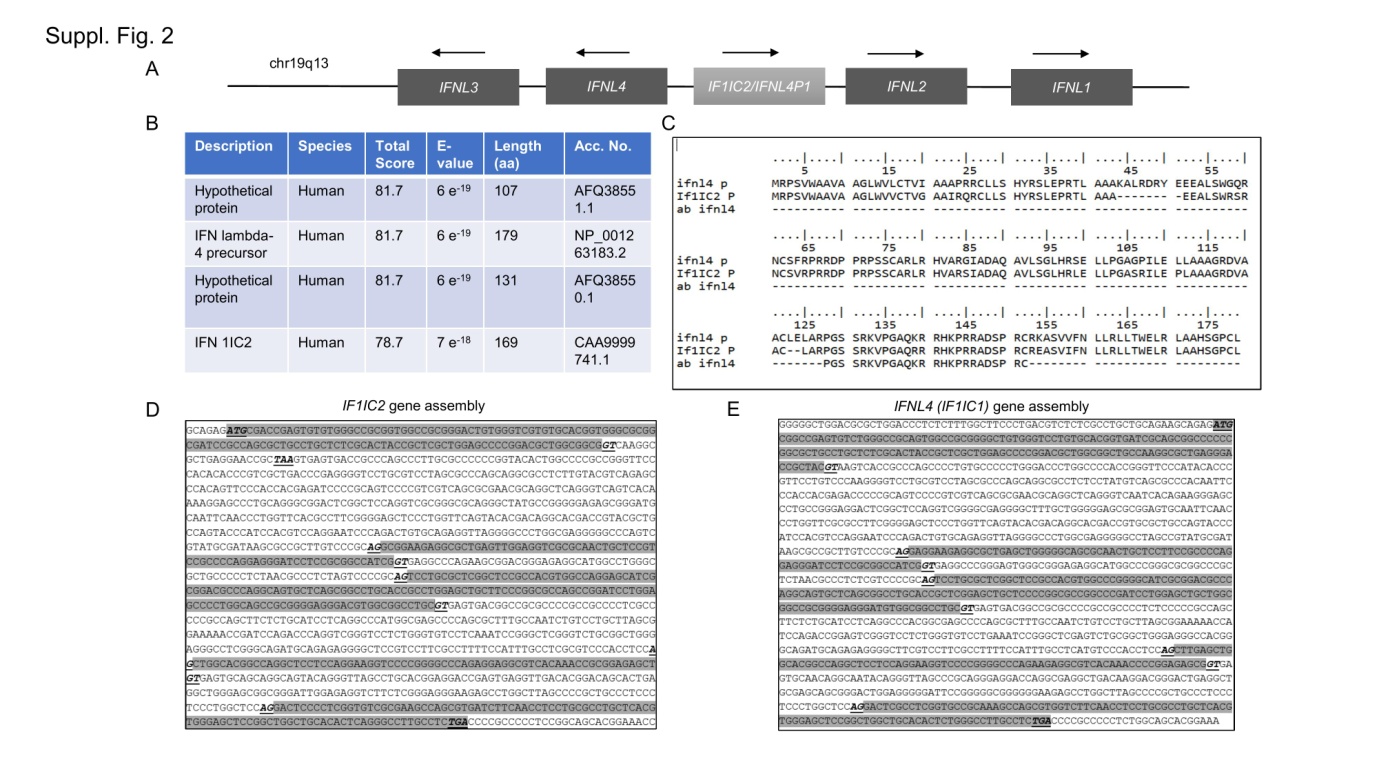

Supplement: Supplemental data [file Suppl_FigureS2.docx]
