# Supplementary material for: A Novel Inactive Isoform with a Restored Reading Frame Is Expressed from the Human Interferon Lambda 4 TT Allele at rs368234815
Source: J Interferon Cytokine Res. 2023 Sep 15;43(9):370–8. doi: 10.1089/jir.2022.0199 (PMC10517323; doi:10.1089/jir.2022.0199)
Supplement: Supplemental data [file Suppl_FigureS3.docx]

**S. Fig. 3**: **A**. WB from Huh7.5 and C33A cells untransfected (UC) or transfected with pcDNA3.1+ (VC) and ΔG allele constructs probed with RAB. HMW-high mol. wt. **B.** WB from HEK293 cells, transfected with pcDNA3.1+ (VC), and cDNA clones of p179: L4-HA-tag, L4-Halo-tag, both transfected at 1ug/well; and ΔG allele constructs, probed with both RAB and MAB. The MAB does not react with the HA-tag version of p179, possibly due to lower abundance than the Halo-tag version. The top schematic shows the binding epitopes of the RAB and MAB within p179. HMW- high mol. wt. **C.** WB from HEK293 cells, transfected with 0.5 ug, 1 ug, and 2 ug/well, of each ΔG and TT, or 1 ug/well of VC (pCDNA+) or pIF1IC2 and probed with MAB. UC-un-transfected cells.

**
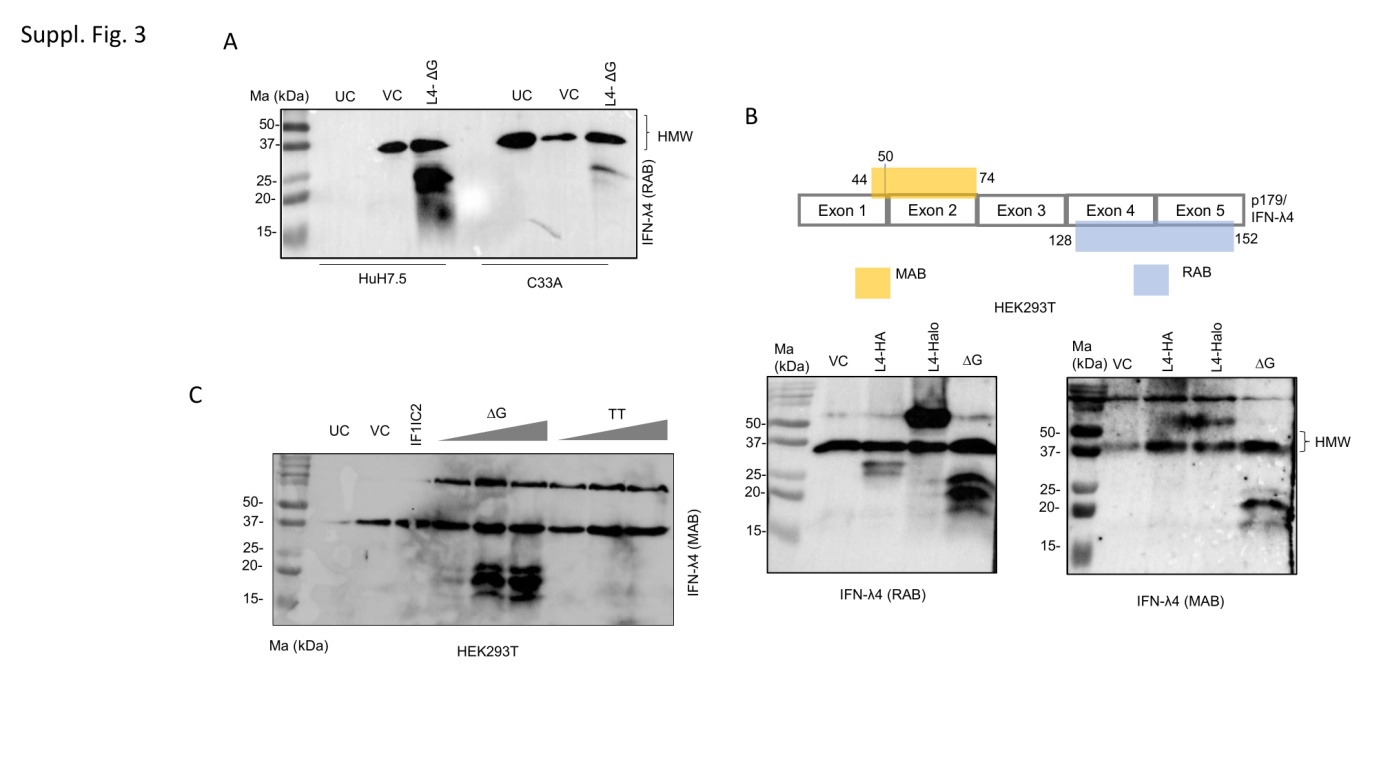
**
