# Supplementary material for: A Novel Inactive Isoform with a Restored Reading Frame Is Expressed from the Human Interferon Lambda 4 TT Allele at rs368234815
Source: J Interferon Cytokine Res. 2023 Sep 15;43(9):370–8. doi: 10.1089/jir.2022.0199 (PMC10517323; doi:10.1089/jir.2022.0199)

**S. Fig. 4**: **A.** WB from C33A and Huh7.5 cells transfected with TT and ΔG, constructs and cell lysates or TCA precipitated supernatants probed with RAB. The secreted protein appeared as a likely aggregate at high molecular size and in a small fraction at the expected molecular size (at ~20 kDa). In Huh7.5 cells, proteins from both the TT and ΔG get secreted unlike in C33A cells where secretion from ΔG is not seen in both the aggregate or monomeric forms. **B .** Schematic representation of all mutations whose results are shown in Fig. 4A, 4D and 3B, and their effect on the open reading frame. **C**. qPCR results for OAS1 gene from HEK293 cells transfected with the different constructs shown. Average of means from two independent experiments carried out in technical triplicates is shown with error bars showing SD of the two averages


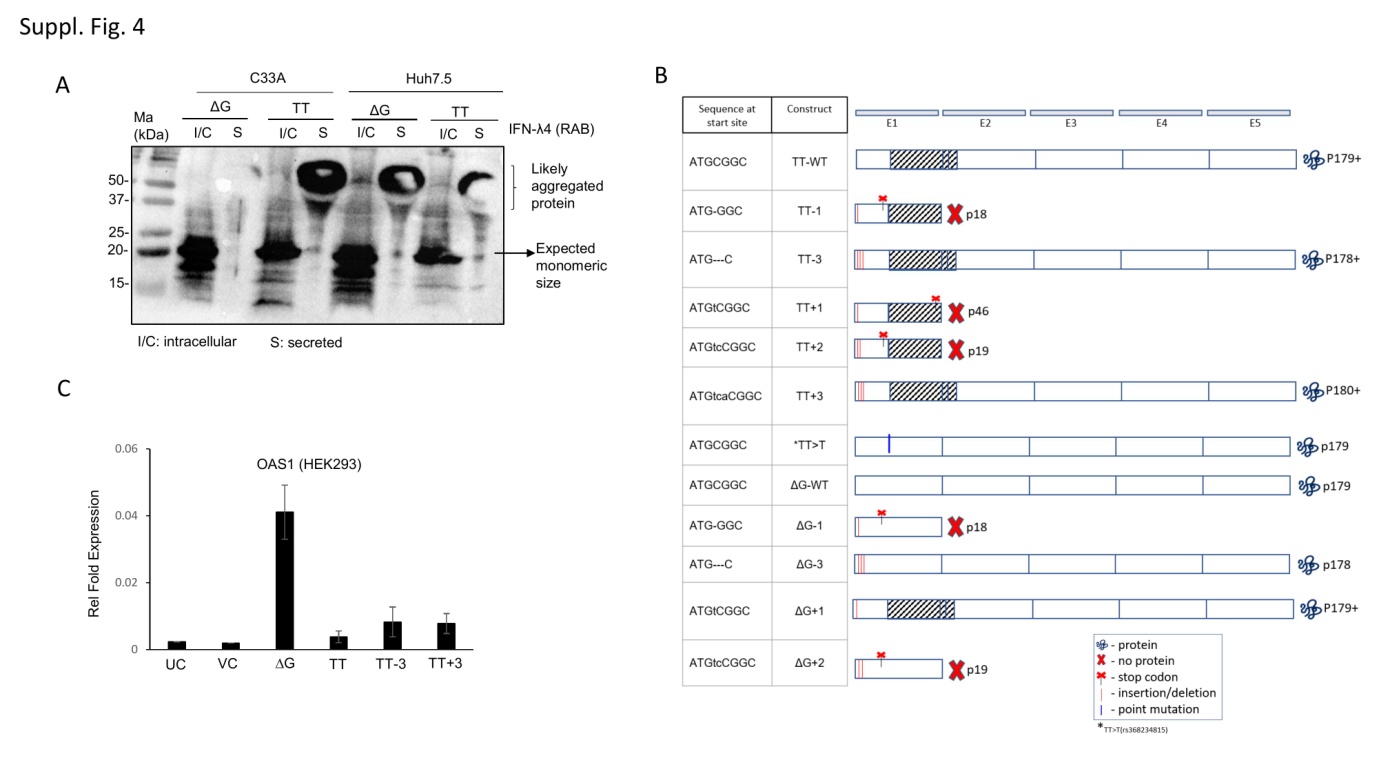

Supplement: Supplemental data [file Suppl_FigureS4.docx]
