# Supplementary material for: A Novel Inactive Isoform with a Restored Reading Frame Is Expressed from the Human Interferon Lambda 4 TT Allele at rs368234815
Source: J Interferon Cytokine Res. 2023 Sep 15;43(9):370–8. doi: 10.1089/jir.2022.0199 (PMC10517323; doi:10.1089/jir.2022.0199)

**S. Fig. 5**: **A.** PCR products amplified from cDNA generated from TT or ΔG allele transfected HEK293 cells using primers that bind to beginning of exon 1 and end of exon 5 (Suppl. Table 1) of the *IFNL4* gene. Different sized amplicons were observed which were gel-purified and the products from the TT allele were sanger sequenced (Bottom table); some products from a different reaction were cloned using TA cloning vectors (Invitrogen) and subsequently sequenced. No new isoforms were identified either by TA cloning or direct sequencing of PCR products. Some overlapping sequences could be seen as smaller peaks in the chromatograms of some sequences of the PCR products suggesting presence of possible alternate spliced isoforms that could not be distinguished by this method. **B**. qPCR was carried out from RNA derived from HEK293 cells transfected with TT or ΔG constructs at 1 ug/well for 12 h; *ACTB* was used as a housekeeping control. The TaqMan primer and probe sets are the same as described in Hong et al, 2016; The qPCR experiment is representative of two independent experiments; mean from one experiment is shown with error bars depicting SD from two technical replicates.


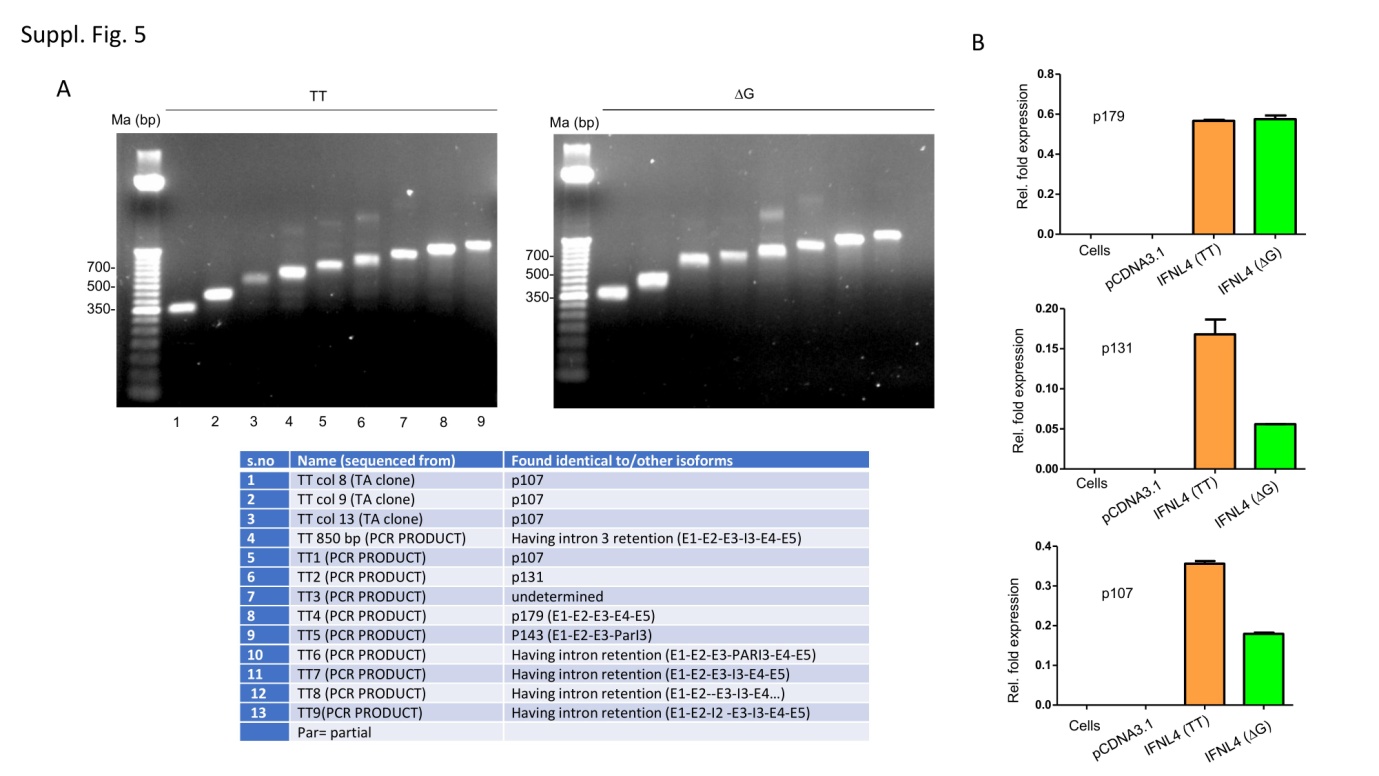

Supplement: Supplemental data [file Suppl_FigureS5.docx]
